# The detour paradigm in animal cognition

**DOI:** 10.1007/s10071-017-1152-0

**Published:** 2017-12-12

**Authors:** Can Kabadayi, Katarzyna Bobrowicz, Mathias Osvath

**Affiliations:** Department of Cognitive Science, Lund University, Helgonavägen 3, 22100 Lund, Sweden

**Keywords:** Detour behavior, Inhibitory control, Route planning, Comparative psychology

## Abstract

In this paper, we review one of the oldest paradigms used in animal cognition: the detour paradigm. The paradigm presents the subject with a situation where a direct route to the goal is blocked and a detour must be made to reach it. Often being an ecologically valid and a versatile tool, the detour paradigm has been used to study diverse cognitive skills like insight, social learning, inhibitory control and route planning. Due to the relative ease of administrating detour tasks, the paradigm has lately been used in large-scale comparative studies in order to investigate the evolution of inhibitory control. Here we review the detour paradigm and some of its cognitive requirements, we identify various ecological and contextual factors that might affect detour performance, we also discuss developmental and neurological underpinnings of detour behaviors, and we suggest some methodological approaches to make species comparisons more robust.

**Electronic supplementary material:**

The online version of this article (10.1007/s10071-017-1152-0) contains supplementary material, which is available to authorized users.

## Introduction

One of the oldest paradigms in animal cognition research involves the use of a see-through obstacle that must be detoured in order to reach the visible goal on the opposite side. Studies on such detour behaviors date back to the beginning of the twentieth century. Hobhouse (1901) tested dogs who could see their owner through a window, and to reach him they had to make a detour and use the door of the house. Thorndike (1911) investigated whether chickens and fish will make roundabouts when a wire or glass barrier blocks the shortest path to a goal room. According to both authors, successful detours exemplify actions that are not solely governed by innate mechanisms because otherwise the animals would head straight for the visible goal.

A decade later, the detour paradigm was employed by the Gestalt psychologist Wolfgang Köhler. He tested chimpanzees, dogs and chickens when they faced a wire fence barrier with a reward on the other side. His aim was to study their potential “insight” capacities. He believed that detours, which required moving away from the goal in order to ultimately reach it, were good tests of whether the goal-directedness was first worked out in the mind (Köhler 1925).

Since then, the detour paradigm has been used in at least 127 studies on at least 96 species, and it has been varied in different ways and used to measure diverse cognitive skills (Table 1, Online Resource 1). The paradigm has also been used in developmental studies on human children, and in neuropsychological studies. In recent years, detour tasks have been employed for large-scale comparative research into the evolution of complex cognitive skills (Kabadayi et al. 2016, 2017a; MacLean et al. 2014). Amidst the ever-growing number of species being tested on various detour tasks, it is time to take stock and look closer at the detour paradigm and the cognitive skills it measures.

**Table 1:** Overview of the cognitive skills measured in the detour paradigm

Tested skills	Explanation
Cognitive and motor development	The execution of reaching and locomotor detours become stabilized throughout infancy, indicating development of inhibitory control and motor development (Bojczyk and Corbetta 2004; Diamond 1990; Lockman 1984)
Functional generalization	Subjects perceive the functional similarity between detour setups that differ in various perceptual features, and they transfer between tasks accordingly (Lockman and Adams 2001)
Inhibitory control	The visible reward behind the barrier creates a strong prepotent tendency for a direct reach. Subjects inhibit this strong perceptual pull and instead execute detour behavior (Diamond 1990)
Insight	A correct solution of the detour problem already on the first trial involves a mental operation where the subject manipulates the problem as a whole and thereafter executes the right response (Köhler 1925; Lorenz 1932)
Learning	Repeated testing of the subjects on detour problems can reveal various learning processes and phenomena such as spatial learning, trial-and-error learning, critical learning period during development, and disruption and retention mechanisms (Fischel 1933; Hull 1938; Scholes 1965; Thorndike 1911)
Social learning	Subjects learn the detour solution by observing another individual demonstrating it (Pongrácz et al. 2005, 2008; Wilkinson et al. 2010)
Task switching	Subjects switch their previously reinforced detour responses if a shortcut option becomes available, and vice versa (Parker et al. 2005; Smith and Litchfield 2010; Thorndike 1911)
Working memory and route planning	When the goal becomes invisible, subjects rely on working memory of the position of the goal, and they plan their detour routes in the absence of perceptual cues emanating from the goal (Cross and Jackson 2016; Wells 1967)

Here we review the detour paradigm within in the field of animal cognition. First, we discuss different types of detour tasks in relation to the cognitive skills they address. We examine various factors that may influence detour behaviors, including ecological, evolutionary and task-specific factors. We also review relevant developmental studies, and those investigating the neurological underpinnings of successful detour behavior. Special attention is paid to the contemporary use of detour tasks as a measure of inhibition. We end with recommendations for future studies.

The review is limited to tasks which require detours to reach a goal behind a barrier when this is *visible* from the animal’s starting position. Following Köhler (1925) and Chapuis (1987), we distinguish between two overarching setups:

- The goal is visible behind the barrier throughout the detour response (*continuously visible goal* detours).
- The goal is initially visible behind the barrier, but it becomes invisible for a certain duration while the animal is moving, due to some added visual occlusions along the way (*initially visible goal* detours).

**Fig. 1:**
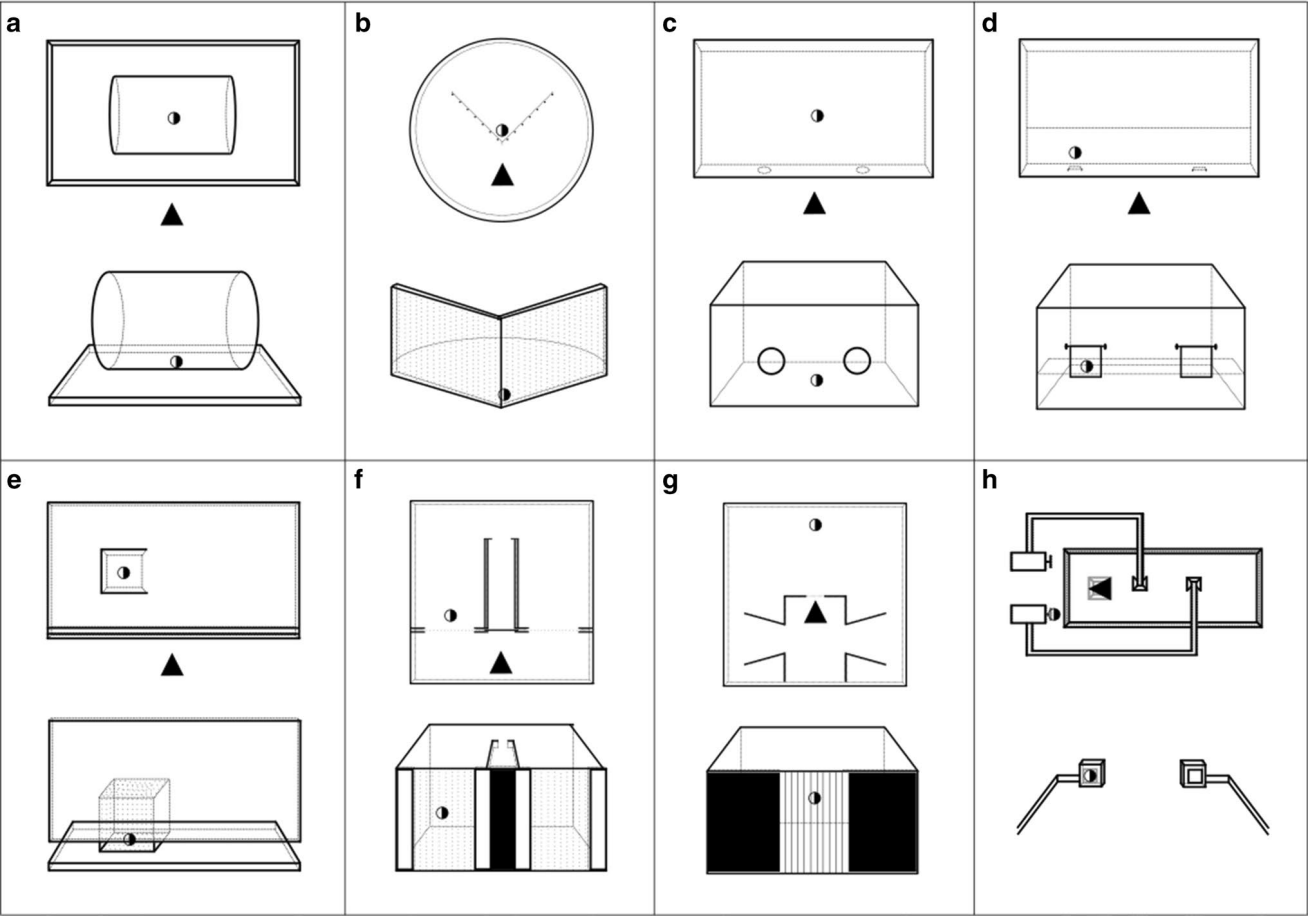
A display of eight of the most common setups in detour tasks; each setup comes with two symbols: a half-filled circle (a goal), and a filled triangle (a subject), and occupies a separate panel. Within each panel, the upper figure shows the bird-eye view, while the lower figure the first-person view. The setups belong to the following tasks: **a** the cylinder task requires a reaching detour through one of the side openings of the transparent cylinder. **b** Inward detour task requires locomotion detour around a V-shaped transparent/fence barrier. **c** Plexiglas-hole task requires a reaching detour through one of the two holes in a Plexiglas panel placed upright between the subject and the reward. **d** Swing-door task requires a reaching detour through the door that is furthest from the goal. The doors can only be opened by pushing them forward, and an attempt to open the door that is closer to the goal results in the goal falling backwards and becoming out of reach. **e** Detour reaching task (object-retrieval task) requires a reaching detour through the side opening of the transparent box. The box has only one opening, which allows changing the open side facing the subject across trials. **f** Delayed-detour task requires passing by an opaque corridor and in the end selecting between two paths, only one of which leads to the goal room. **g** Four-compartment box task requires the subject to turn its back to the goal and choose among four doors, only two of which lead to the goal room. **h** Detour-choice task requires the subject to turn its back to the goal that is placed on top of a tower, and select among two poles, only one of which leads to the tower where the goal is placed

Although different detour tests share an underlying characteristic—the direct path to the goal is blocked and a roundabout way must be taken—they also differ considerably in their perceptual features (Fig. 1). Locomotor detours often require the subject to move its entire body around the barrier, whereas reaching detours require a reach only with a limb. Moreover, the barriers come in different shapes (U-, V-, I-, L-, J-shaped) and materials. Some barriers are fully transparent (e.g., glass), and some are semitransparent (e.g., fence/mesh). Semitransparent barriers can in turn have horizontal or vertical grid patterns, or both (Online Resource 1). Variation in such perceptual features often affects the detour performance (Table 2).

**Table 2:** Several factors that may affect the detour performance

Factor	Examples
Distance to the goal	Increased goal distance makes it easier to execute detours (Diamond 1990; Junghans et al. 2016; Köhler 1925; Regolin et al. 1994)
Neophobia	Animals tend to perform worse when they are in a fearful state, e.g., when tested alone in a novel environment (Regolin et al. 1995)
Orientation of the barrier	In V-shaped barriers, dogs and goats perform better detours when they have to take outward detours compared to inward detours (Nawroth et al. 2016; Pongracz et al. 2001). Chickens execute better detours with horizontal grid pattern fence barriers compared to vertical grid ones, likely because the vertical grid barriers offer better goal visibility for a moving animal (Regolin et al. 1994)
Previous experience with transparent objects	Since the transparent objects are artificial, animals lacking previous experience with them might initially fail on the transparent barrier tasks because they did not learn that they cannot pass through transparent material. It takes experience to resolve the tactile-visual conflict presented with the transparent barriers (Bojczyk and Corbetta 2004)
Rearing conditions	Data suggest that animals raised in enriched environments perform better in detour tasks compared to those raised in low enriched environments (Clarke et al. 1951)
Reward visibility	The increased visibility of the reward behind the barrier creates a perceptual pull for a direct reach, thus making it harder to execute detour response (Chapuis et al. 1983; Juszczak and Miller 2016; Lockman 1984; Lockman and Adams 2001; Poucet et al. 1983; Regolin et al. 1994; Zucca et al. 2005)
Motivation	Type of the reward behind the barrier as well as the motivational and physiological state of the animal can affect the detour response (Regolin et al. 1995; Shaw 2017)
Age	Younger individuals might fail on detour tasks due to cognitive/motor immaturity (Diamond 1990; Lockman 1984)

## Cognitive mechanisms investigated with the detour paradigm

### Inhibitory control

Köhler (1925, p.11) begins the first chapter of his influential book, The Mentality of the Apes, with the following paragraph: “When any of those higher animals, which make use of vision, notice food (or any other objective) somewhere in their field of vision, they tend—so long as no complications arise—to go after it in a straight line. We may assume that this conduct is determined without any previous experience, providing only that their nerves and muscles are mature enough to carry it out.” To contrast such behaviors, he then describes situations where a direct way to the target is blocked, and the animal thus must make a detour to reach the goal. Such situations would require some mental operations beyond innate responses.

Köhler (1925) described his observations on dogs, chickens and chimpanzees making detours around wire fences. Whereas dogs and chimpanzees were usually successful, chickens had difficulties and often attempted to go directly for the food through the fence. But he also found individual variation within the species, and later studies have shown remarkable detour performances in chickens, even within few days after hatching (Regolin et al. 1994, 1995; Scholes 1965; Scholes and Wheaton 1966).

As Köhler notes, the goal visibility behind the barrier is one of the major factors influencing detour performance. A common finding in detour studies is that detours become harder to perform if the goal behind the barrier is more clearly visible, e.g., when the occlusion is reduced through mesh grids or Plexiglas barriers (chickens: Regolin et al. 1994; dogs: Brucks et al. 2017; human infants: Lockman and Adams 2001; mice: Juszczak and Miller 2016, ring doves and pigeons: Miller and Tallarico 1974). Some researchers argue the visible reward behind the barrier acts like a “perceptual magnet,” creating a prepotent tendency for a direct reach (Vallortigara and Regolin 2002). This makes it difficult to move away from the visible goal as a detour requires. In line with this, many species are better in making detours around opaque barriers compared to almost identical but transparent ones (cats: Poucet et al. 1983; chickens: Regolin et al. 1994; dogs: Chapuis et al. 1983; human infants: Lockman 1984, Noland 2008; mice: Juszczak and Miller 2016, although see Osthaus et al. 2010).

Similar to goal visibility, the distance to the goal affects detour behaviors: with increasing goal distance, it becomes easier to execute detours (chickens: Regolin et al. 1995; dogs: Köhler 1925; human infants: Diamond and Gilbert 1989; long-tailed macaques: Junghans et al. 2016; toads: Lock and Collett 1979). This suggests a closer goal creates a stronger lure which makes it harder to move away from. The effect of goal distance on detours has also been the subject of various theoretical analyses on detour behavior (Hull 1938; Lewin 1933; Tolman 1932; reviewed in: Rashotte 1987).

Such findings have led to the interpretation of detour tasks as a measure of executive functions, and more precisely of behavioral/motor inhibition: the subject must inhibit the predominant motor response of directly reaching for the reward and instead make a detour (Diamond 1990, 1991; Moll and Kuypers 1977). The fact that most subjects execute efficient detours around opaque barriers while having problems with identical but transparent barriers suggests a knowing/acting mismatch that is common to other inhibition tasks: the subjects know the detour solution; however, they cannot act on the knowledge because the visible reward creates a strong lure for a direct reach, thus bumping into the barrier (Diamond 2013).

Detour tasks have been used to study inhibitory control in development (see “The development of detour behaviors” section), in neurocognition (see “Neurological underpinnings of detour behaviors” section), and in inter-species comparisons (see “Ecological factors” section). The relative practical ease of using such tasks has begun to turn detour tasks into one of the benchmark tests of inhibition in inter-species comparisons and phylogenetic reconstructions (Amici et al. 2008; Kabadayi et al. 2016; MacLean et al. 2014; Vlamings et al. 2010). However, there is an ongoing discussion of the interpretation of the results concerning the vast sensorimotor variation among the tested species, numerous non-cognitive contextual factors that may affect performance (Table 2), and the learning/ceiling effects sometimes seen over trials (see “Learning” section).

In general, detour tasks, which are used to measure inhibitory control, belong to *continuously visible goal* detours, where the goal is visible behind the barrier throughout the detour response (Fig. 1a–e). For example, reaching detours belong to this category, where the subject uses its limb to retrieve a goal from the side opening of a transparent box or cylinder (Diamond 1990; MacLean et al. 2014, Fig. 1a, e). Performances in such visible conditions are sometimes juxtaposed with non-visible conditions (opaque apparatuses), in order to control for the effect of goal visibility (Diamond 1990; Lockman 1984).

### Working memory and route planning

Even though *initially visible goal* detour setups can be used for testing inhibition, they are more suitable for studying working memory and route planning. In such tasks, the animals arguably form some sort of a mental representation of the goal position, which they then keep in mind for a given amount of time when the goal goes out of sight.

As described, in *initially visible goal* detour tasks the goal behind the barrier is visible from the starting position, but becomes out of view when the animal moves. This is usually achieved by opaque walls along the detour path or in some parts of the barrier itself (Fig. 1f). A majority of such detour setups also require a choice between multiple routes, only a subset of which lead to the goal (Fig. 1f–h). During this selection, the animals cannot see the goal.

For example, octopuses (*Octopus vulgaris*) have been tested in such design, also known as a delayed-detour test (Schiller 1949a, b; Wells 1964, 1967, 1970). In these experiments, the rewards were visible behind a barrier from the starting position. To reach the reward, the animals had to move forward into an opaque corridor, and choose between two openings at the exit, with only one opening leading to the reward room (Fig. 1f). The octopuses successfully completed around three out of every four trials.

Similarly, jumping spiders have been tested on *initially visible goal* detour tasks, where the individual, from the starting position, could see a distant goal placed on top of a pole (Fig. 1h). A direct jump was not possible, and only a roundabout walking route—without the reward in sight—led to the goal; a binary route choice was again available to the individual (Cross and Jackson 2016; Tarsitano and Jackson 1997). The spiders were often highly successful in these tasks (see “Ecological factors” section).

Several *initially visible goal* detour studies also made use of a four-compartment box; after spotting the goal behind a transparent/semitransparent barrier, the individual had to turn away from the barrier and head toward one of the four opaque compartments, with only two leading to the goal (chickens: Regolin et al. 1995; canaries, herring gulls and quails: Zucca et al. 2005, Fig. 1g).

Successful responses in such tasks have usually been interpreted as a result of the animal’s mental representation of the non-visible goal (Vallortigara and Regolin 2002), and its use of such representations in planning the detour routes (Cross and Jackson 2016). However, no agreement has been reached on the nature of these representations, and their very existence has been questioned (Barrett 2011; Cross and Jackson 2016). For example, although some interpret the results as cases of planning where the motor response is preceded by a decision (Cross and Jackson 2016), others argue the detour behaviors emerge from step-wise and situated processes, in which the animal uses environmental and sensorimotor affordances, such as wall following or visual scanning (jumping spiders: Barrett 2011; Tarsitano 2006; octopus: Wells 1967).

 Support for the embodied perspective comes from robotics, showing that robots succeed in detours not by representing the out-of-“sight” goal position, but rather by exploratory behavior using sensory feedback and the physical constraints in the environment (Walker and Miglino 1999). The setup used in this study replicated Regolin et al. (1995) who tested chickens. After initially “seeing” the goal behind the barrier, the robot had to turn its back to the reward and select from four opaque compartments, two of which led to the goal (Fig. 1g). The performance of robots was similar to the chickens’ (22 out of 24 robots chose the right compartments), despite the lack of preprogrammed maps or other internal representations of the position of the goal (Miglino et al. 1998; Ponticorvo et al. 2007; Walker and Miglino 1999). Obviously, these results cannot tell us whether chickens use mental representations when solving the task, but only that it is in principle not necessary.

The behavior of the animal at the choice point in *initially visible goal* detours might be especially relevant in studying the process of deliberation and planning. At similar choice points where only one route leads to an out-of-sight goal, rats seemingly deliberate over their choices in a process called vicarious trial-and-error, where they pause and look back and forth before they chose a path (Redish 2016). During vicarious trial-and-error, the hippocampal place cells encode future outcomes (Johnson and Redish 2007) where the animal seemingly deliberates over the future alternatives. Similar neurological and behavioral investigations at choice points on *initially visible goal* detour tasks might shed light on the possible involvement of similar processes such as prospection in solving these detour tasks. Since vicarious trial-and-error occurs mostly when the animal faces the problem for the first time, and disappears after repeated trials (Redish 2016), it is advisable to avoid repeated trials per individual with *initially visible goal* detour tasks to capture this process of deliberation.

### Learning

In most detour studies, individuals have been tested repeatedly, and several studies have found improvements over trials (Beniuc 1938; Boogert et al. 2011; Burghardt 1964; de Haan 1949; Fischel 1933; Lorenz 1932; Parker et al. 2005, 2012; Schiller 1949a, b; Scholes 1965; Scholes and Wheaton 1966; Smith and Litchfield 2010; Spigel 1964; Thorndike 1911; Vernouillet et al. 2016; Vlamings et al. 2010; Wallis et al. 2001; Wyrwicka 1959; however, see: Pongracz et al. 2001, 2008; Wells 1967; Zucca et al. 2005). Similarly, many studies have found animals become faster over trials in reaching the goal around the barrier, indicating yet another learning effect (Beniuc 1938; Burghardt 1964; Lockman and Adams 2001; Parker et al. 2005; Spigel 1964; Thorndike 1911; Wyrwicka 1959; but see Baragli et al. 2011 for a lack of such effect).

Accordingly, detour tasks were employed to study various learning processes. For example, studies focusing on critical learning periods compared the rate of improvement on detour tasks across different age groups in early development to explore the peak learning period (chickens: Scholes 1965, Scholes and Wheaton 1966). Other studies used detour tasks to explore learning and retention/disruption mechanisms (European green lizards: Fischel 1933; fish: Thorndike 1911; painted turtles: Spigel 1964). Research on social learning investigated whether some animals learn to solve detour problems through observing other individuals executing the detour behavior (Pongrácz et al. 2005, 2008; Wilkinson et al. 2010).

Hull (1938) offered a model based on stimulus–response (S-R) learning in explaining the gradual improvement of detour behavior. According to this model, such an improvement happens because the excitatory potential of the direct pathway is gradually reduced due to repeated failed attempts and barrier touches. The detour behavior emerges when the excitatory strength of the indirect pathway becomes stronger than the direct pathway (Rashotte 1987; see also Wyrwicka 1959). A similar model was offered from the field of robotics to explain detour behavior based on reactive problem solving (Balkenius 1994).

In detours involving visible rewards, the recruited cognitive mechanisms might differ between when solving the problem for the first time and after repeated trials. As mentioned, Köhler believed detour behaviors, especially when performed on the first trial, signaled insightful behavior as the animal must perceive the whole problem ahead of the detour (Köhler 1925). Some detour studies, mostly *initially visible goal* detours, have followed a strict one trial method, to explore whether animals spontaneously solve a detour problem (Atkinson 2003; Cross and Jackson 2016; Köhler 1925; Regolin et al. 1994, 1995; Regolin and Rose 1999; Sun et al. 2010; Tarsitano and Andrew 1999; Tarsitano and Jackson 1994, 1997; Zucca et al. 2005).

Successful detours already in the first trial versus gradual increase of successes across trials are often distinguished and labeled differently: detour behavior versus detour learning (Vallortigara and Regolin 2002); spatial reasoning versus spatial learning or trial-and-error learning (Smith and Litchfield 2010; Wynne and Leguet 2004). Trial-and-error learning of the detour problem suggests a possibility that successfully solving the detour problem might arise from chance. One can imagine that after an initial frustration resulting from multiple bumps into the barrier, the animal gives up and walks away from the goal; then, once it reaches the end of the barrier, it follows the visual, acoustic or olfactory cues from the goal to finally reach it (Scholes 1965; Vallortigara and Regolin 2002). To reduce the chance that a detour occurs by chance, some studies have used modifications, mostly by offering animals multiple options (Atkinson 2003; Regolin et al. 1995; Tarsitano and Jackson 1994; Zucca et al. 2005).

Köhler suggested one should observe the movement pattern of the animal in order to distinguish whether the detour results from a thoughtful process or from chance (Köhler 1925). He argued that in the case of the “real” solution, the movement of the animal displays unity in time and space: from the starting position the animal directly goes for a reward in a single line without hesitation. Conversely, solutions resulting from chance are often characterized by the sum of disparate and discontinuous movements where the animal zigzags in front of the barrier; and only once it has seen the reward from outside the barrier its movements become continuous toward the reward. Such a mechanism based on chance was also central for the S-R theories in explaining the initial solution to the detour problem (Hull 1938; Rashotte 1987).

However, seemingly contradicting the behavioristic stance (Hull 1938), learning effects do not only come gradually, but sometimes also rapidly after the first successful detour solution (Beniuc 1938; Regolin et al. 1995; Regolin and Rose 1999; Siniscalchi et al. 2013). Regolin et al. (1995) claimed that instead of gradual learning, rapid improvement may emerge from overcoming stress responses due to being tested in a novel environment. Besides, learning cannot explain all successful detour performances in studies that have used repeated trials as some species did perform well already from the initial trials (Bray et al. 2014; Kabadayi et al. 2016; MacLean et al. 2014; Marshall-Pescini et al. 2015; Smith and Litchfield 2010).

### Task switching

On the assumption that some detour tasks measure inhibition, swift improvement of the performance across trials might be inconsistent with findings from other inhibition tasks where no or very little improvement over trials is found (Berkman et al. 2014; Zelazo et al. 1996). Moreover, in contrast to other motor inhibition tasks, individuals often reach and maintain a ceiling level of perfect accuracy in detour tasks after repeated testing (song sparrows: Boogert et al. 2011; parrots: Kabadayi et al. 2017a). In classical behavioral inhibition tasks, subjects know the task rules but are unable to follow them to achieve and preserve peak performance even after being tested repeatedly, because the task presents additional inhibitory challenges, often due to task switching. For example, on certain trials subjects have to inhibit an already initiated response (stop-signal task) or a response that has been repeated previously (go/no-go task). However, one can achieve and preserve peak performance in detour tasks by following the same strategy that proved to be effective in the previous trial(s). Neurological evidence suggests the detour task may cease to measure inhibition after the task is acquired and perfect accuracy is reached (Walker et al. 2006). This suggests after repeated trials with detour tasks, the knowing/acting mismatch weakens, and the visual reward behind the barrier no longer exerts a strong pull on direct reaching behavior.

Offering shortcuts on certain trials might test whether the detour response becomes habitual after repeated trials (Verbruggen et al. 2014). This idea was already implemented over 100 years ago by Thorndike, who found if chickens used a detour path in around 75–80 trials, they tended to ignore shortcuts that were later made available (Thorndike 1911). Similarly, dogs appear to have difficulty using shortcuts after detouring over repeated trials. They tend to cling onto the detour response instead of taking the shorter path, suggesting functional fixedness (Pongracz et al. 2003a, b). The tendency of repeating the old—but not the most appropriate—response is especially pronounced if the dogs learn the detour from a human, perhaps as a result of domestication (Pongracz et al. 2003a, b). Likewise, dogs commit preservative errors by sticking to the previously reinforced detour despite an explicit change in the detour setup that requires a different response (Clarke et al. 1951; Hobhouse 1901; Osthaus et al. 2010).

 Shortcuts could also be used in a task-switching context and increase the inhibitory requirements by adding a learned component that must be inhibited (Monsell 2003). The general setup in the few studies that have used this task-switching component is the offering of a shortcut through an opening in the barrier; and after the animals have used the shortcut over repeated trials, removing the shortcut thereby forces the animals to take a detour. Whereas using the shortcut significantly deteriorates the subsequent detour response for dogs (Marshall-Pescini et al. 2015; Pongracz et al. 2003a, b), dingoes show pronounced task-switching skills as they detour equally efficiently around the barrier after the shortcut is no longer available (Smith and Litchfield 2010). Similarly, squirrel monkeys have more difficulties in making detours after the shortcut is blocked (Lyons et al. 2000; Parker et al. 2005, 2012), suggesting a cost from task switching (Monsell 2003). Implementing shortcuts in detour setup prevents a ceiling effect of perfect accuracy (Jentsch et al. 2000; Parker et al. 2005, 2012). Transparent detour apparatuses with only one opening (Fig. 1e) are suitable for such task-switching problems as one can change the open side that faces the subject across the trials (Lyons et al. 2000; Parker et al. 2012).

### Functional generalization

Are different versions of detour tasks understood by animals in similar ways because they share the same underlying principle? Such functional generalization would allow an animal to take into account higher-order functional aspects of a detour problem and ignore irrelevant perceptual features (Call 2013; Jacobs and Osvath 2015).

Evidence suggests that perceptual features might considerably affect detour performance in many species (see Table 2). For example, children found it more challenging to make locomotor detours than reaching detours around transparent barriers (Lockman and Adams 2001). Similarly, a study found no correlation between performances of dogs and wolves in equivalent locomotor and reaching detour tasks (Marshall-Pescini et al. 2015). The orientation of the barrier also affects the success of chickens, dogs and goats (Nawroth et al. 2016; Pongracz et al. 2001; Regolin et al. 1994). With V-shaped barriers, both dogs and goats were more successful in outward (subject begins inside the vertex of V) than inward (subject begins outside the vertex of V) detours (Nawroth et al. 2016; Pongracz et al. 2001, Table 2, Fig. 1b).

The lack of generalization of different detour problems can also be explained from an action–perception perspective where the subjects, through their interactions with the barriers, obtain information about the specific affordances and possibilities for action, and they are not merely reacting to reward visibility (Lockman and Adams 2001; Thelen et al. 2001). However, this does not mean all species lack functional generalization when it comes to detour tasks. Such generalization might help when faced with a transparent barrier after training on an identical but opaque barrier, as is often done in many studies (e.g., MacLean et al. 2014; Wallis et al. 2001). Those species that are more efficient in perceiving the functional similarity between opaque and transparent barriers should find it easier to detour around the transparent barrier. In primates for example, the lateral prefrontal cortex seems to mediate this ability of functional generalization and task transfer between opaque and transparent barriers (Wallis et al. 2001, see “Neurological underpinnings of detour behaviors” section).

## Ecological, neurological and developmental underpinnings of detour behaviors

### Ecological factors

As many species face problems similar to the detour paradigm in their environments, detour tests likely often reflect ecologically relevant situations, and detour problems might be more ecologically meaningful for some species compared to others. Various comparative studies using detour problems attributed the results partly to the ecology of the tested species (birds: Miller and Tallarico 1974; Zucca et al. 2005; canines: Marshall-Pescini et al. 2015; Pongracz et al. 2001; Smith and Litchfield 2010; great apes: Vlamings et al. 2010; jumping spiders: Cross and Jackson 2016; Tarsitano and Jackson 1994; monkeys: Amici et al. 2008; reptiles: Burghardt 1977).

For example, the perception of the task features might differ between species due to their ecology. Dogs find it harder to take inward detours rather than outward detours around V-shaped barriers, probably because they tend to avoid constricted spaces (Pongracz et al. 2001). Analogously, how obstacles are perceived may differ between aerial and terrestrial species: vertical obstacles may be more ecologically meaningful for flying species compared to earth bound ones (Lorenz 1971). This may explain why chickens, which are poor flyers, performed worse on detours around barriers with vertical bars than with horizontal ones (Regolin et al. 1994; Vallortigara and Regolin 2002), whereas herring gulls displayed an opposite pattern (Zucca et al. 2005). Similarly, Zucca et al. (2005) found that canaries performed markedly worse in a detour problem (Fig. 1g) compared to quails and young herring gulls. The authors suggested the adaptation to terrestrial or aerial habitats could explain this difference: whereas in their natural environment canaries could avoid the detour problem simply by flying, the detour problem was ecologically meaningful for ground-living quails and young herring gulls that use walking as the main mode of locomotion.

Similarly, detour problems might be more ecologically meaningful for predators compared to prey species, as their pursuit of prey often involves detours around obstacles (Lorenz 1932). For example, jumping spiders performed remarkably well in many detour problems (Cross and Jackson 2016); and this was attributed to their ecology as in the wild they navigate complex three-dimensional environments when searching for prey, using their well-developed visual system (Tarsitano 2006).

In general, certain skills, that are adaptive within a certain ecological niche, might prove more useful than others in solving various detour problems. Thus, ecological factors must be addressed both in the task’s design and in the interpretation of the results. Some authors have given central importance to such ecological factors. For example, Burghardt (1977) asserted that detour behaviors are as much a result of the dominant sense, and cue relevance and species ecology, as of “intelligence.” However, hardwired predispositions due to species-specific ecological factors cannot solely explain detour success, as there are learning effects and individual variation within species (Dettmer et al. 2015; Frank and Frank 1982; Juszczak and Miller 2016; Köhler 1925). Due to these differences, an average success rate in the task is often not sufficient in inter-species comparisons; instead, a variance of the species-specific success rates may be more informative.

Others have taken a more integrated approach, using detour tasks across species to measure the socio-ecological correlates of certain cognitive skills. For example, Vlamings et al. (2010) found orangutans outperformed chimpanzees, bonobos and gorillas in a detour task called the swing-door task, which is considered to test for inhibition (Fig. 1d). The authors partly attributed the high inhibitory skills of orangutans to the reduced food competition among group mates compared to other great ape species. Similarly, Amici et al. (2008) used two detour tasks—the swing-door task and Plexiglas-hole task (Fig. 1c)—as parts of a task battery measuring inhibitory control in seven species of primates, and found that the species living in more dynamic and fluid social environments (fission–fusion societies) outperformed those having more cohesive group structures. The authors concluded primates living in more complex social groups often require inhibition of inappropriate prepotent responses in a dynamic social environment, and this partly explains why they performed better in detour tasks.

Attempts to find tasks that could be applicable to wide range of species have led to a simplification of the detour setup. More recently, a detour task was designed utilizing a hollow transparent cylinder and it was named “the cylinder task” (Bray et al. 2014). This task requires inhibition of a direct reach for the reward placed centrally inside a hollow transparent cylinder, and an execution of a detour through one of the side openings instead (Fig. 1a). Each subject receives training on an opaque cylinder before being tested on a transparent, but otherwise identical, cylinder. The training ensures the subjects learn the correct detour solution; then, the subsequent errors in the transparent condition may be attributed to the inhibition failure (Santos et al. 1999), consistent with the idea of the existence of a knowing/acting mismatch. The cylinder task was recently administered to 36 different species—29 mammal and seven bird species—in order to study the evolution of motor inhibition (MacLean et al. 2014). This study found that the great apes were the most successful on the cylinder task of all the species tested and that absolute brain size significantly correlated with the task performance (see “Neurological underpinnings of detour behaviors” section).

### Neurological underpinnings of detour behaviors

Most neurocognitive studies have relied on induced lesions in primate brains to find correlations between various brain regions and the execution of effective detour responses. The object-retrieval task, a detour reaching task around a transparent cubicle (Fig. 1e), is the most popular detour task used in these lesion studies, and successful performance in this task has been interpreted as an expression of inhibitory control. The lesion studies targeted the prefrontal cortex, given its role in supporting inhibitory processes (Diamond 1990).

Rhesus monkeys with dorsolateral prefrontal cortex ablations and marmoset monkeys with joint lesions of orbitofrontal and lateral prefrontal cortex had difficulties with detouring transparent barriers (Diamond and Goldman-Rakic 1985; Dias et al. 1996; Moll and Kuypers 1977). Other studies on African green monkeys have suggested low dopamine levels in the striatum and the prefrontal cortex, as well as serotonin depletions in orbitofrontal cortex, deteriorate the detour performance around transparent barriers (Jentsch et al. 1997, 1999a, b, 2000; Taylor et al. 1990a, b; Walker et al. 2006).

A study on marmoset monkeys suggested two separate and dissociable systems play a role for detours around transparent barriers (Wallis et al. 2001). Orbitofrontal cortex lesioned marmosets performed poorly compared to controls in detours around a transparent box. But after extensive training with an identical but an opaque box, the lesioned monkeys overcame their problems. In contrast, lateral prefrontal cortex lesioned monkeys had problems transferring the detour behavior they learned in the opaque box to the transparent box. This suggests at least two different brain areas facilitate detours around transparent barriers: one for motor inhibition (the orbitofrontal cortex) and another for task transfer between the opaque and the transparent barrier (the lateral prefrontal cortex). This study also provides a neurological explanation for the common behavioral finding that experience with opaque barriers improve detours around transparent, but otherwise identical, barriers (Juszczak and Miller 2016; Santos et al. 1999).

 A recent large-scale comparative study testing 36 species, 29 mammal and 7 bird species found absolute brain size strongly predicted detour performance on the cylinder task (Fig. 1a), with great apes as the best performers (MacLean et al. 2014). This conclusion was soon challenged when three corvid species (ravens, New Caledonian crows and jackdaws) showed scores similar to great apes on the cylinder task, despite having vastly smaller absolute brains sizes (Kabadayi et al. 2016). However, corvids have much greater neural densities than primates (Olkowicz et al. 2016), and it has been suggested that total number of pallial neurons is a better predictor of cognitive ability—including cylinder task performance—than absolute brain size (Herculano-Houzel 2017). But parrots, despite having similarly high numbers of pallial neurons, performed poorly on the cylinder task (Kabadayi et al. 2017a). Such discrepant findings suggest the level of analyses should focus on specific brain regions rather than the whole pallium. As mentioned, in primates, detours around the transparent barriers are mediated by prefrontal regions. In birds, the associative brain area called nidopallium caudolateral (NCL) is an obvious candidate for a similar function. The NCL functions analogously to the mammalian prefrontal cortex (Güntürkün 2005) and mediates other executive processes such as working memory as well as motor inhibition (Kalt et al. 1999; Veit and Nieder 2013).

### The development of detour behaviors

Comparative developmental studies using detour tasks may also provide insights into the evolution of cognition. Cognitive developmental research investigates the emergence of adaptive systems from the various combinations of cognitive building blocks during development (Gómez 2005). Development is a key evolutionary mechanism, and developmental investigations can complement comparative studies in order to reach a better understanding of cognitive evolution (Rosati et al. 2014). For example, they can reveal if different species attain similar cognitive skills using similar or different building blocks (Osvath et al. 2014). Despite this potential importance, there are few comparative developmental studies, with rhesus monkeys and ravens representing the only non-human species tested longitudinally through development (Diamond 1990; Kabadayi et al. 2017b).

Detour problems with transparent or fence barriers have been used to study the development of inhibitory skills and motor control in human infants (Bojczyk and Corbetta 2004; Diamond 1990; Lockman 1984; Lockman and Adams 2001; Piaget 1954) and rhesus monkey infants (Diamond and Goldman-Rakic 1986). Human infants exhibit a clear developmental progression between the sixth and the 12th month of life in detouring transparent barriers (Diamond 1981; Diamond and Gilbert 1989), and similar developmental trajectory has been found in infant rhesus monkeys between the first and the fourth month (Diamond and Goldman-Rakic 1986). During a certain period, both human and rhesus monkey infants perform better with an opaque barrier compared to an identical but transparent one (Diamond 1981, 1990; Lockman 1984). This “opaque advantage” during a certain developmental period suggests the problem with detours around transparent barriers is one of inhibition. The gradual overcoming of the difficulty of detouring around transparent barriers has thus been attributed to the development of executive functions and to the maturation of prefrontal cortex (Diamond 1990, 1991; however, see below for a different view from the perspective of motor control).

Raven chicks have similar difficulties during development in inhibiting a direct reach for a reward behind a transparent barrier, but they overcome this difficulty and succeed in the cylinder task when they are around 10 weeks old (Kabadayi et al. 2017b). Before the 10th week, and after they attain object permanence, they perform better on opaque barriers than transparent ones. Their detour performance on a fence barrier is better than on a fully transparent barrier. This suggests reduced reward visibility makes it easier to inhibit motor responses (for a similar finding in human infants: Lockman and Adams 2001; Noland 2008).

Developmental studies can also demarcate the relative contributions of motor experience and cognitive maturation in solving detour problems. For example, Diamond (1988) found human infants tested longitudinally (tested every second week) succeeded on the detour reaching task around 2–4 weeks earlier than infants in the cross-sectional group [for a similar finding in ravens see Kabadayi et al. (2017b)]. This suggests repeated motor experience with the task helps infants solve the task earlier, and the successful detours develop not solely from a task-independent inhibitory maturation, but also from an interaction of various processes such as sensorimotor experience with the barrier as well as motor coordination (Williams et al. 2015).

## Conclusion and future directions

There are numerous advantages of using the detour paradigm. It is easy to administer and often ecologically meaningful: many animals face situations in their natural environment where various obstacles block the shortest path to the goal. However, there is surprisingly little agreement on the cognitive requirements for successful detours. Inhibitory control is a common interpretation of *continuously visible goal* detours; however, rule learning and functional generalization/task transfer are other cognitive skills likely involved in solving detour problems. Instead of comparing the average scores obtained over a fixed number of trials on detour tasks, investigating how species obtain those scores might yield better understanding on these other cognitive processes involved in solving the task and hence lead to a more robust comparison of the cognitive skills between species (Güntürkün et al. 2017).

For example, increasing the number of training trials received with an opaque cylinder might shed light to the relative contribution of functional generalization to the cylinder task performance. Similarly, measuring the change in latency to obtain a reward behind a barrier might reveal an operant/rule learning component. Inserting shortcuts into the detour setup can measure whether the detour response has become habitual after repeated trials, and implementing switches between shortcuts and detours may measure task-switching skills. Systematically targeting these different cognitive processes by controls and modifications in detour setups can allow robust quantification of cognitive traits, which would lead to meaningful intra-and inter-species comparisons (Thornton et al. 2014).

The executive function interpretation of the detour problem is based on the knowing/acting mismatch: the subject must have the necessary physical knowledge to solve the detour problem, and yet it cannot reflect this knowledge in action because the visible reward behind the barrier creates a lure and the subject acts impulsively by attempting a direct reach. The inhibitory faculty thus liberates the already existing knowledge from the intrusion of impulsive tendencies. Thus, studies focusing on inhibition should ensure the animals tested know the impenetrability of the transparent barrier. This means they should have sufficient experience with transparent objects before being tested with transparent barrier detour tasks. This is especially important given that transparent objects are highly artificial (animals rarely face transparent barriers in their natural environment) and present conflicting visual/tactile information. Inhibition tasks often infer costs when choosing the immediate gratification/acting impulsively. However, some detour tasks—such as the cylinder task—do not infer a major cost to animals when they make a contact with the barrier in an attempt to directly reach for the reward. In order to make inhibitory failures costlier, one can modify the detour setup, e.g., a direct contact to the barrier makes the reward unavailable, as in the swing-door task (Fig. 1d, also see: Hughes and Russell 1993).

When it comes to *initially visible goal* detours, there is a similar dispute concerning the precise nature of the representations necessary to solve such tasks. The core question depends on whether animals plan their detour routes at the beginning of the task when they can see the reward, and later use those representations when choosing the correct pathway among alternatives, when the reward goes out of sight. Controlling for factors such as landmark use and path integration is helpful to test this question. Another promising avenue might be to observe the behavior, and possibly the brain, at the choice point: a vicarious trial-and-error behavior and an accompanying future-encoding place cell activity might reflect a process of deliberation and planning (Redish 2016). First trials are of importance for such observations because the deliberation process tends to disappear after repeated trials (Redish 2016).

Neurological correlates of detour behavior might provide useful information on the cognitive mechanisms measured by detour tasks. For example, neuropsychological studies focused on specific brain regions suggested that for primates, *visible goal* detour tasks measure not only inhibition, but also functional generalization (Wallis et al. 2001); and the detour task may stop measuring inhibition once the ceiling level of perfect accuracy is reached (Walker et al. 2006). Consistently, future comparative studies should focus on specific brain regions, instead of broad correlations such as absolute/relative brain size and the total number of pallial neurons.

Comparative developmental studies can provide useful tools to approach cognitive evolution from an ontogenical perspective, i.e., how cognition emerges from various constellations of cognitive building blocks (Gómez 2005). Thus, developmental comparisons may reveal novel patterns that cannot be uncovered by comparing adult performances (Rosati et al. 2014). Similar developmental patterns between different species suggest a similarly constructed cognitive skill, whereas differences in pattern may reveal a different architecture, which can explain differences in mature cognition. There are only few developmental studies on detour behaviors. Expanding such developmental studies would be promising in answering whether the pace and pattern of development share similarities across lineages (Rosati et al. 2014).

We have pointed toward various contextual factors that affect detour performances (Table 2). Comparative studies should take into account such factors for robust comparisons. Simplifying the task is a convenient attempt in this direction, but there are still numerous factors that should be controlled for, such as the previous experience with transparency, motivation, size and material of the barrier, age and sensorimotor capability of the animal. Since task-specific factors may favor certain species at the expense of the others, avoiding single tasks and using detour task batteries might lead to more robust comparisons.

## Electronic supplementary material

Below is the link to the electronic supplementary material.

Supplementary material 1 (DOCX 67 kb)
