# Supplementary material for: The detour paradigm in animal cognition
Source: Anim Cogn. 2017 Dec 12;21(1):21–35. doi: 10.1007/s10071-017-1152-0 (PMC5756264; doi:10.1007/s10071-017-1152-0)
Supplement: Supplementary file 1 — Supplementary material 1 (DOCX 67 kb) [file 10071_2017_1152_MOESM1_ESM.docx]

**Online Resource 1:** Overview of the empirical studies with the species tested, different detour setups and the cognitive skills measured

| Species | Task | Goal visibility | | Response | | | Barrier | | | | Capacity measured | | | References | |  |
| --- | --- | --- | --- | --- | --- | --- | --- | --- | --- | --- | --- | --- | --- | --- | --- | --- |
|  |  |  |  |  |  |  | **Transparency** | | **Shape** | |  |  |  |  |  |  |
| Apes (*Hominoidae*) | | | | | | | | | | |  |  |  |  |  |  |
| Bonobo  (*Pan paniscus*) | plexiglas-hole task | continuous | | reach | | | transparent | | I | | inhibitory control | | | Amici et al 2008 | |  |
|  | swing-door task | continuous | | reach | | | transparent | | I | | inhibitory control | | | Amici et al 2008,  Vlamings et al 2010 | |  |
|  | cylinder task | continuous | | reach | | | transparent | | cylinder | | inhibitory control | | | MacLean et al 2014 | |  |
| Chimpanzee  (*Pan trolodytes*) | plexiglas-hole task | continuous | | reach | | | transparent | | I | | inhibitory control | | | Amici et al 2008 | |  |
|  | swing-door task | continuous | | reach | | | transparent | | I | | inhibitory control | | | Amici et al 2008,  Vlamings et al 2010 | |  |
|  | cylinder task | continuous | | reach | | | transparent | | cylinder | | inhibitory control | | | MacLean et al 2014 | |  |
|  | retrieving an object thrown through a window | initial | | locomotion | | | transparent | | irregular | | insight, memory | | | Köhler 1925 | |  |
| Western gorilla  (*Gorilla gorilla*) | plexiglas-hole task | continuous | | reach | | | transparent | | I | | inhibitory control | | | Amici et al 2008 | |  |
|  | swing-door task | continuous | | reach | | | transparent | | I | | inhibitory control | | | Amici et al 2008,  Vlamings et al 2010 | |  |
|  | cylinder task | continuous | | reach | | | transparent | | cylinder | | inhibitory control | | | MacLean et al 2014 | |  |
| Human  (*Homo sapiens*) | roundabout | continuous | | reach | | | transparent | | I | | motor development, functional generalization | | | Lockman 1984,  Lockman & Adams 2001 | |  |
| Human  (*Homo sapiens*) | roundabout | continuous | | locomotion | | | transparent | | I | | motor development, functional generalization | | | Lockman 1984,  Lockman & Adams 2001 | |  |
|  |  | continuous | | reach | | | semitransparent | | I | | motor development, functional generalization | | | Lockman & Adams 2001 | |  |
|  |  | continuous | | locomotion | | | semitransparent | | I | | motor development, functional generalization | | | Lockman & Adams 2001 | |  |
|  | swing-door task | continuous | | reach | | | transparent | | I | | inhibitory control | | | Vlamings et al 2010 | |  |
|  | object retrieval task | continuous | | reach | | | transparent | | cubicle | | cognitive development | | | Diamond 1981, Diamond & Gilbert 1989, Bell & Fox 1992, Matthews et al 1996 | |  |
|  |  | continuous | | reach | | | semitransparent | | I | | motor development | | | Bojczyk & Corbetta 2004 | |  |
|  | object retrieval task | continuous | | reach | | | transparent | | I | | inhibitory control | | | Noland 2008,  Noland & Rodrigues 2012 | |  |
|  |  | continuous | | reach | | | semitransparent | | I | | inhibitory control | | | Noland 2008 | |  |
|  | detour reaching task | continuous | | reach | | | transparent | | box | | inhibitory control, task switching | | | Hughes & Russell 1993, Hughes et al 1998,  Edgin et al 2008 | |  |
| Bornean orangutan  (*Pongo pygmaeus*) | plexiglas-hole task | continuous | | reach | | | transparent | | I | | inhibitory control | | | Amici et al 2008 | |  |
|  | swing-door task | continuous | | reach | | | transparent | | I | | inhibitory control | | | Amici et al 2008  Vlamings et al 2010 | |  |
|  | cylinder task | continuous | | reach | | | transparent | | cylinder | | inhibitory control | | | MacLean et al 2014 | |  |
| Old World monkeys (*Cercopithecoidae*) | | | | | | | | | | | | | | | |  |
| African green monkey (*Cercopithecus aethiops sabaeus*) | detour reaching task | continuous | | reach | | | transparent | | cubicle | | inhibitory control,  task switching | | | Taylor et al 1990ab, Jentsch et al 1997, Jentsch et al 1999ab, Jentsch et al 2000, Elsworth et al 2012, Olausson et al 2007 | |  |
| Golden monkey (*Cercopithecus kandti*) | cylinder task | continuous | | reach | | | transparent | | cylinder | | inhibitory control | | | MacLean et al 2014 | |  |
| Hamadrayas baboon (*Papio hamadryas*) | cylinder task | continuous | | reach | | | transparent | | cylinder | | inhibitory control | | | MacLean et al 2014 | |  |
| Long-tailed macaque (*Macaca fascicularis*) | plexiglas-hole task | continuous | | reach | | | transparent | | I | | inhibitory control | | | Amici et al 2008, Junghans et al 2016 | |  |
|  | swing-door task | continuous | | reach | | | transparent | | I | | inhibitory control | | | Amici et al 2008 | |  |
|  | object retrieval task | continuous | | reach | | | transparent | | cubicle | | cognitive development | | | Diamond et al 1989 | |  |
|  |  | continuous | | reach | | | transparent | | cubicle | | inhibitory control | | | Schneider & Pope-Coleman 1995, Schneider et al 1998, Rutten et al 2008, Sutcliffe et al 2014 | |  |
| Olive baboon  (*Papio anubis*) | cylinder task | continuous | | reach | | | transparent | | cylinder | | inhibitory control | | | MacLean et al 2014 | |  |
| Pig-tailed macaque (*Macaca nemestrina*) | object retrieval task | continuous | | reach | | | transparent | | cubicle | | inhibitory control | | | Schneider & Roeltgen 1993, Gray et al 2006 | |  |
| Rhesus macaque (*Macaca mulatta*) | cylinder task | continuous | | reach | | | transparent | | cylinder | | inhibitory control | | | MacLean et al 2014 | |  |
|  | object retrieval task | continuous | | reach | | | transparent | | cubicle | | cognitive development | | | Diamond & Goldman-Rakic 1985,1986 | |  |
|  |  | continuous | | reach | | | transparent | | cubicle | | Inhibitory control | | | Cannon et al 2013,  Smith et al 2013, Eddins et al. 2014, Uslaner et al 2013 | |  |
| Rhesus macaque (*Macaca mulatta*) | detour reaching task | continuous | | reach | | | transparent | | floor plate | | inhibitory control | | | Moll & Kuypers 1977 | |  |
|  |  | continuous | | reach | | | transparent | | cubicle | | inhibitory control, task switching | | | Dettmer et al 2015 | |  |
| New World monkeys (*Platyrrhini*) | | | | | | | | | | | | | | | |  |
| Capuchin monkey (*Cebus apella*) | plexiglas-hole task | continuous | | reach | | | transparent | | I | | inhibitory control | | | Amici et al 2008 | |  |
|  | swing-door task | continuous | | reach | | | transparent | | I | | inhibitory control | | | Amici et al 2008 | |  |
|  | cylinder task | continuous | | reach | | | transparent | | cylinder | | inhibitory control | | | MacLean et al 2014 | |  |
|  | detour reaching task | continuous | | reach | | | transparent | | cubicle | | inhibitory control | | | Roitberg et al 2002 | |  |
|  | object retrieval task | continuous | | reach | | | transparent | | cubicle | | Inhibitory control | | | Lakshminarayan & Santos 2009 | |  |
| Common marmoset (*Callithrix jacchus*) | cylinder task | continuous | | reach | | | transparent | | cylinder | | inhibitory control | | | MacLean et al 2014 | |  |
|  | object retrieval task | continuous | | reach | | | transparent | | cubicle | | inhibitory control | | | Dias et al 1996, Wallis et al 2001, Walker et al 2006, Murai et al 2013, Pryce et al 2004 | |  |
| Cotton-top tamarin  (*Saguinus oedipus)* | object retrieval task | continuous | | reach | | | transparent | | cubicle | | inhibitory control | | | Santos et al 1999 | |  |
| Geoffroy's spider monkey (*Ateles geoffroyi)* | plexiglas-hole task | continuous | | reach | | | transparent | | I | | inhibitory control | | | Amici et al 2008 | |  |
|  | swing-door task | continuous | | reach | | | transparent | | I | | inhibitory control | | | Amici et al 2008 | |  |
| Golden-headed lion tamarin  (*Leontopithecus chrysomelas*) | cylinder task | continuous | | reach | | | transparent | | cylinder | | inhibitory control | | | MacLean et al 2014 | |  |
| Squirrel monkey  (*Saimiri sciureus*) | cylinder task | continuous | | reach | | | transparent | | cylinder | | inhibitory control | | | MacLean et al 2014 | |  |
|  | detour reaching task | continuous | | reach | | | transparent | | cubicle | | inhibitory control, task switching | | | Lyons et al 2000, Parker et al 2005, 2012 | |  |
| Lemuriform primates (*Strepsirrhini*) | | | | | | | | | | | | | | | |  |
| Ave ave (*Daubentonia madagascariensis*) | cylinder task | continuous | | reach | | | transparent | | cylinder | | inhibitory control | | | MacLean et al 2014 | |  |
| Black lemur  (*Eulemur macaco)* | cylinder task | continuous | | reach | | | transparent | | cylinder | | inhibitory control | | | MacLean et al 2014 | |  |
| Brown lemur  (Eulemur fulvus) | cylinder task | continuous | | reach | | | transparent | | cylinder | | inhibitory control | | | MacLean et al 2014 | |  |
| Coquerel’s sifaka (*Prophitecus coquereli)* | cylinder task | continuous | | reach | | | transparent | | cylinder | | inhibitory control | | | MacLean et al 2014 | |  |
| Mongoose lemur  (*Eulemur mongoz*) | cylinder task | continuous | | reach | | | transparent | | cylinder | | inhibitory control | | | MacLean et al 2014 | |  |
| Red-bellied lemur  (*Eulemur rubriventer)* | cylinder task | continuous | | reach | | | transparent | | cylinder | | inhibitory control | | | MacLean et al 2014 | |  |
| Ring-tailed lemur (*Lemur kata*) | cylinder task | continuous | | reach | | | transparent | | cylinder | | inhibitory control | | | MacLean et al 2014 | |  |
| Ruffed lemur  (*Varecia variegata*) | cylinder task | continuous | | reach | | | transparent | | cylinder | | inhibitory control | | | MacLean et al 2014 | |  |
| Canids (*Canidae*) | | | | | | | | | | |  |  |  |  |  |  |
| Coyote  (*Canis latrans*) | cylinder task | continuous | | reach | | | transparent | | cylinder | | inhibitory control | | | MacLean et al 2014 | |  |
| Dingo (*Canis latrans*) | inward detour | continuous | | locomotion | | | semitransparent | | V | | task switching, social learning | | | Smith & Litchfield 2010 | |  |
| Dog  (*Canis familiaris)* | roundabout | continuous | | locomotion | | | semitransparent | | L | | insight | | | Köhler 1925 | |  |
|  |  | continuous | | locomotion | | | semitransparent | | I | | problem solving | | | Sarris 1937, Thompson & Heron 1954 | |  |
|  |  | continuous | | locomotion | | | semitransparent | | I, irregular | | associative learning | | | Wyrwicka 1959 | |  |
|  |  | continuous | | locomotion | | | semitransparent | | I | | motor response learning | | | Osthaus et al 2010 | |  |
|  |  | continuous | | locomotion | | | semitransparent | | I | | route planning | | | Chapuis et al 1983 | |  |
|  |  | initial | | locomotion | | | semitransparent | | I | | insight | | | Köhler 1925 | |  |
|  |  | initial | | locomotion | | | semitransparent | | U | | problem solving | | | Clarke et al 1951 | |  |
|  | inward detour | continuous | | locomotion | | | semitransparent | | V | | problem solving | | | Sarris 1937 | |  |
|  |  | continuous | | locomotion | | | semitransparent | | V | | social learning | | | Pongracz et al 2001, 2003ab, 2005, 2008 | |  |
|  |  | continuous | | locomotion | | | transparent | | V | | inhibitory control | | | Bray et al 2015 | |  |
|  |  | continuous | | locomotion | | | semitransparent | | V | | inhibitory control | | | Marshall-Pescini et al 2015 | |  |
|  | outward detour | continuous | | locomotion | | | semitransparent | | V | | problem solving | | | Sarris 1937 | |  |
|  | outward detour | continuous | | locomotion | | | semitransparent | | U, I | | problem solving | | | Scott & Fuller 1965 | |  |
|  |  | continuous | | locomotion | | | semitransparent | | V | | social learning | | | Pongracz et al 2001, 2003b | |  |
|  |  | initial | | locomotion | | | semitransparent | | V | | lateralization | | | Siniscalchi et al 2013 | |  |
| Dog  (*Canis familiaris)* | cylinder task | continuous | | locomotion, reach | | | transparent | | cylinder | | inhibitory control | | | Bray et al 2014, MacLean et al 2014, Marshall-Pescini et al 2015, Fagnani et al. 2016 | |  |
|  | detour reaching task | continuous | | locomotion, reach | | | transparent | | box | | inhibitory control | | | Brucks et al. 2017 | |  |
|  | retrieving an object thrown through a window | initial | | locomotion | | | transparent | | irregular | | insight, memory | | | Köhler 1925 | |  |
| Gray wolf  (*Canis lupus*) | inward detour | continuous | | locomotion | | | semitransparent | | V | | inhibitory control | | | Marshall-Pescini et al 2015 | |  |
|  | outward detour | continuous | | locomotion | | | semitransparent | | U, I | | problem solving | | | Frank & Frank 1982 | |  |
|  | cylinder task | continuous | | locomotion, reach | | | transparent | | cylinder | | inhibitory control | | | MacLean et al 2014, Marshall-Pescini et al 2015 | |  |
| Equids (*Equidae*) | | | | | | | | | | |  |  |  |  |  |  |
| Donkey (*Equus asinus)* | roundabout | continuous | | locomotion | | | semitransparent | | I | | motor response learning | | | Osthaus et al 2013 | |  |
| Horse  (*Equinus caballus*) | roundabout | continuous | | locomotion | | | no obstacle  at eye level | | I | | lateralization | | | Murphy et al 2005 | |  |
|  |  | continuous | | locomotion | | | semitransparent | | I | | motor response learning | | | Osthaus et al 2013 | |  |
| Horse  (*Equinus caballus*) | roundabout | continuous | | locomotion | | | semitransparent | | U | | social learning | | | Dalla Costa et al 2013 | |  |
|  |  | continuous | | locomotion | | | no obstacle  at eye level | | I | | motor response learning | | | Savin & Randle 2013 | |  |
|  | inward detour | continuous | | locomotion | | | semitransparent | | U | | social learning | | | Rorvang et al 2015 | |  |
|  | outward detour | continuous | | locomotion | | | no obstacle  at eye level | | U | | lateralization | | | Baragli et al 2011 | |  |
| Mule (*Equus caballus ×  Equus asinus*) | roundabout | continuous | | locomotion | | | semitransparent | | I | | motor response learning | | | Osthaus et al 2013 | |  |
| Other mammals | | | | | | | | | | | | | | | |  |
| Cat  (*Felis catus)* | roundabout | continuous | | locomotion | | | semitransparent | | I | | route planning | | | Poucet et al 1983 | |  |
|  | delayed detour task | initial | | locomotion | | | semitransparent | | irregular | | working memory | | | Schiller 1950 | |  |
| Goat  (*Capra hircus*) | inward detour | continuous | | locomotion | | semitransparent | | | V | | social learning | | Nawroth et al 2016 |  |  |  |
| Fox squirrel (*Sciurus niger*) | cylinder task | continuous | | locomotion, reach | | | transparent | | cylinder | | inhibitory control | | | MacLean et al 2014 | |  |
| Mongolian gerbil (*Meriones unguiculatus)* | cylinder task | continuous | | locomotion, reach | | | transparent | | cylinder | | inhibitory control | | | MacLean et al 2014 | |  |
| Mouse (F1 hybrid) | inward detour | continuous | | locomotion | | | transparent | | V | | inhibitory control | | | Juszczak & Miller 2016 | |  |
|  |  | continuous | | locomotion | | | semitransparent | | V | | inhibitory control | | | Juszczak & Miller 2016 | |  |
|  | outward detour | continuous | | locomotion | | | transparent | | V | | inhibitory control | | | Juszczak & Miller 2016 | |  |
| Mouse (F1 hybrid) | outward detour | continuous | | locomotion | | | semitransparent | | V | | inhibitory control | | | Juszczak & Miller 2016 | |  |
| Quokka  (*Setonix brachyurus*) | roundabout | continuous | | locomotion | | | semitransparent | | L | | lateralization, spatial learning | | | Wynne & Leguet 2004 | |  |
| Sheep (*Ovis aries*) | roundabout | continuous | | locomotion | | | no obstacle  at eye level | | I | | lateralization | | | Versace et al 2007 | |  |
| Birds *(Aves)* | | | | | | | | | | | | | | | |  |
| Budgerigar (*Melopsittacus undulatus*) | cylinder task | | continuous | | locomotion, reach | | | transparent | | cylinder | | problem solving | | | Kemp 2016 | |
| Canary  (*Serinus canaria*) | four-compartment box | | initial | | locomotion | | | transparent | | I | | working memory | | | Zucca et al 2005 | |
|  |  |  | initial | | locomotion | | | semitransparent | | I, vertical-bar, horizontal-bar | | working memory | | | Zucca et al 2005 | |
| Canary  (*Serinus canaria*) | four-compartment box | | initial | | locomotion | | | semitransparent | | I, small-grid, large-grid | | working memory | | | Zucca et al 2005 | |
| Clark’s nutcracker (*Nucifraga columbiana)* | cylinder task | | continuous | | locomotion, reach | | | transparent | | cylinder | | inhibitory control | | | Vernouillet et al 2016 | |
| Chicken (*Gallus gallus domesticus)* | roundabout | | continuous | | locomotion | | | semitransparent | | I | | intelligence | | | Thorndike 1911 | |
|  |  |  | continuous | | locomotion | | | semitransparent | | L | | insight | | | Köhler 1925 | |
|  |  |  | initial | | locomotion | | | transparent | | U | | spatial navigation | | | Regolin et al 1994 | |
|  |  |  | initial | | locomotion | | | semitransparent | | U, vertical-bar, horizontal-bar | | spatial navigation | | | Regolin et al 1994 | |
|  |  |  | initial | | locomotion | | | semitransparent | | U, small-grid, large-grid | | spatial navigation | | | Regolin et al 1994 | |
|  | roundabout | | initial | | locomotion | | | semitransparent | | U, I | | spatial navigation | | | Regolin & Rose 1999 | |
|  |  |  | initial | | locomotion | | | transparent | | H | | spatial learning | | | Sun et al 2010 | |
|  | outward detour | | continuous | | locomotion | | | semitransparent | | U | | insight | | | Köhler 1925 | |
|  | four-compartment box | | initial | | locomotion | | | semitransparent | | I | | spatial learning | | | Regolin et al 1995 | |
| Chicken (*Gallus gallus domesticus)* | detour through a tunnel | | initial | | locomotion | | | transparent | | I | | critical learning period during development | | | Scholes 1965 | |
|  |  |  | initial | | locomotion | | | transparent | | I | | critical learning period during development | | | Scholes & Wheaton 1966 | |
| Eurasian jay (*Garrulus glandarius)* | cylinder task | | continuous | | locomotion, reach | | | transparent | | cylinder | | inhibitory control | | | MacLean et al 2014 | |
| Herrling gull (*Larus cachinnans)* | four-compartment box | | initial | | locomotion | | | transparent | | I | | working memory | | | Zucca et al 2005 | |
|  |  |  | initial | | locomotion | | | semitransparent | | I, vertical-bar, horizontal-bar | | working memory | | | Zucca et al 2005 | |
|  |  |  | initial | | locomotion | | | semitransparent | | I, small-grid, large-grid | | working memory | | | Zucca et al 2005 | |
| Jackdaw  (*Corvus monedula*) | roundabout | | initial | | locomotion | | | semitransparent | | irregular | | problem solving | | | Lorenz 1932 | |
|  | cylinder task | | continuous | | locomotion, reach | | | transparent | | cylinder | | inhibitory control | | | Kabadayi et al 2016 | |
| Japanese quail (*Coturnix japonica)* | roundabout | | continuous | | locomotion | | | semitransparent | | L, I | | spatial learning | | | Kiker et al 1976 | |
|  | roundabout | | continuous | | locomotion | | | semitransparent | | I, grid-bar | | lateralization | | | Zucca & Sovrano 2008 | |
| Japanese quail (*Coturnix japonica)* | four-compartment box | | initial | | locomotion | | | transparent | | I | | working memory | | | Zucca et al 2005 | |
|  |  |  | initial | | locomotion | | | semitransparent | | I, vertical-bar, horizontal-bar | | working memory | | | Zucca et al 2005 | |
|  |  |  | initial | | locomotion | | | semitransparent | | I, small-grid, large-grid | | working memory | | | Zucca et al 2005 | |
| New Caledonian crow (*Corvus moneduloides)* | cylinder task | | continuous | | locomotion, reach | | | transparent | | cylinder | | inhibitory control | | | Kabadayi et al 2016 | |
| North island robin (*Petroica longipes*) | cylinder task | | continuous | | locomotion, reach | | | transparent | | cylinder | | Inhibitory control | | | Shaw 2017, Shaw et al. 2015 | |
| Orange-winged amazon (*Amazona amazonica*) | cylinder task | | continuous | | locomotion, reach | | | transparent | | cylinder | | inhibitory control | | | MacLean et al 2014 | |
| Raven  (*Corvus corax*) | cylinder task | | continuous | | locomotion, reach | | | transparent | | cylinder | | inhibitory control | | | Kabadayi et al 2016 | |
| Ring dove  (*Streptopelia risoria*) | roundabout | | continuous | | locomotion | | | semitransparent | | I | | problem solving | | | Miller & Tallarico 1974 | |
| Song sparrow (*Melospiza melodia*) | cylinder task | | continuous | | locomotion, reach | | | transparent | | cylinder | | inhibitory control | | | Boogert et al 2011, MacLean et al 2014 | |
| Sulphur-crested cockatoo (*Cacatua galerita*) | roundabout | | continuous | | locomotion | | | semitransparent | | I | | problem solving, insight | | | Lorenz 1932 | |
| Swamp sparrow (*Melospiza georgiana)* | cylinder task | | continuous | | locomotion, reach | | | transparent | | cylinder | | inhibitory control | | | MacLean et al 2014 | |
| Western scrub-jay (*Aphelocoma californica*) | cylinder task | | continuous | | locomotion, reach | | | transparent | | cylinder | | inhibitory control | | | MacLean et al 2014 | |
| White Carneau pigeon (*Columba livia)* | cylinder task | | continuous | | locomotion, reach | | | transparent | | cylinder | | inhibitory control | | | MacLean et al 2014 | |
|  | roundabout | | continuous | | locomotion | | | semitransparent | | I | | problem solving | | | Miller & Tallarico 1974 | |
| Zebra finch (*Taeniopygia guttata)* | cylinder task | | continuous | | locomotion, reach | | | transparent | | cylinder | | inhibitory control | | | MacLean et al 2014 | |
| Reptiles *(Reptilia)* |  |  | |  | | |  | |  | |  | | |  | |  |
| European green lizard (*Lacerta viridis*) | roundabout | continuous | | locomotion | | | semitransparent | | I | | learning | | | Fischel 1933 | |  |
| Common chameleon (*Chamaeleo chamaeleon*) | outward detour | initial | | locomotion | | | transparent | | U | | lateralization | | | Lustig et al 2013 | |  |
| Painted turtle (*Chrysemys picta*) | roundabout | initial | | locomotion | | | semitransparent | | U | | learning | | | Spigel 1964 | |  |
| Red-footed tortoise (*Geochelone carbonaria*) | inward detour | continuous | | locomotion | | | semitransparent | | V | | social learning | | | Wilkinson et al 2010 | |  |
| Amphibians *(Amphibia)* | | | | | | | | | | |  |  |  |  |  |  |
| European green toad (*Bufo viridis)* | inward detour | initial | | locomotion | | | semitransparent | | U | | route planning, task switching | | | Collett 1982 | |  |
| Northern leopard frog  (*Rana pipiens)* | roundabout | continuous | | locomotion | | | semitransparent | | I | | behavioral discrimination  of line direction | | | Ingle 1971 | |  |
| Cane toad  (*Bufo marinus*) | roundabout | continuous | | locomotion | | | semitransparent | | I | | spatial learning | | | Lock & Collett 1979 | |  |
|  | roundabout | initial | | locomotion | | | semitransparent | | I | | spatial learning | | | Lock & Collett 1979 | |  |
| Neotropical frog (*Allobetes femoralis*) | escape tunnel task | initial | | locomotion | | | void | | irregular | | Spatial learning | | | Munteanu et al 2016 | |  |
| Fish *(Pisces)* | | | | | | | | | | |  |  |  |  |  |  |
| Cardinal Brachy (*Brachyrhaphis roseni*) | roundabout | continuous | | locomotion | | | semitransparent | | I | | lateralization | | | Bisazza et al 1997 | |  |
| Convict cichlid (*Amatitlania nigrofasciata*) | roundabout | continuous | | locomotion | | | semitransparent | | I | | lateralization | | | Moscicki et al 2011 | |  |
| Eastern mosquitofish (*Gambusia holbrooki*) | roundabout | continuous | | locomotion | | | semitransparent | | I | | lateralization | | | Bisazza et al 1997 | |  |
| Fighting fish (*Betta splendens*) | roundabout | continuous | | locomotion | | | transparent | | I | | spatial learning | | | Beniuc 1938 | |  |
| Goldbelly topminnow (*Girardinus falcatus*) | roundabout | continuous | | locomotion | | | semitransparent | | I | | lateralization | | | Bisazza et al 1997 | |  |
|  |  | continuous | | locomotion | | | semitransparent | | I, vertical-bar | | lateralization | | | Facchin et al 1999 | |  |
|  | detour-choice task | continuous | | locomotion | | | semitransparent | | I, vertical-bar | | lateralization | | | Bisazza et al 1998 | |  |
| Guppy (*Poecilia reticulata*) | roundabout | continuous | | locomotion | | | semitransparent | | I | | lateralization | | | Bisazza et al 1997 | |  |
|  | cylinder task | continuous | | locomotion | | | transparent | | cylinder | | inhibitory control | | | Lucon–Xiccato et al 2017 | |  |
|  | roundabout | continuous | | locomotion | | | transparent | | I | | inhibitory control | | | Lucon–Xiccato et al 2017 | |  |
| Minnow (*Phoxinus laevis*) | delayed detour task | continuous | | locomotion | | | transparent | | irregular | | working memory | | | Schiller 1948 | |  |
| Nicaraguan mosquitofish (*Gambusia nicaraguensis*) | roundabout | continuous | | locomotion | | | semitransparent | | I | | lateralization | | | Bisazza et al 1997 | |  |
| Pearl cichlid  (*Geophagus brasiliensis*) | roundabout | continuous | | locomotion | | | semitransparent | | I | | lateralization | | | Reddon et al 2009 | |  |
| Fish (unspecific) | a box with partitions | continuous | | locomotion | | | transparent | | I | | intelligence | | | Thorndike 1911 | |  |
|  |  | continuous | | locomotion | | | semitransparent | | I | | intelligence | | | Thorndike 1911 | |  |
| Molluscs *(Mollusca)* | | | | | | | | | | |  |  |  |  |  |  |
| Flamed tigersnail (*Anguispira alternata*) | roundabout | continuous | | locomotion | | | semitransparent | | I | | problem solving | | | Shearer & Atkinson 2001 | |  |
| Flamed tigersnail (*Anguispira alternata*) | roundabout | continuous | | locomotion | | | semitransparent | | J | | problem solving | | | Atkinson 2003 | |  |
|  | outward detour | continuous | | locomotion | | | semitransparent | | V | | problem solving | | | Atkinson 2003 | |  |
| Octopus  (*Octopus vulgaris*) | roundabout | continuous | | locomotion | | | semitransparent | | I | | problem solving | | | de Haan 1949 | |  |
|  | delayed detour task | initial | | locomotion | | | semitransparent | | irregular | | working memory | | | Schiller 1949 | |  |
|  |  | initial | | locomotion | | | transparent | | irregular | | working memory | | | Wells 1964, 1967, 1970 | |  |
| Salticids *(Salticidae)* | | | | | | | | | | | | | | | |  |
| Algerian jumping spider (*Cyrba algerina*) | detour-choice task | initial | | locomotion | | | void | | n/a | | spatial navigation, route planning | | | Cross & Jackson 2016 | |  |
| Black-headed  jumping spider (*Trite planiceps*) | detour-choice task | continuous | | locomotion | | | void | | n/a | | spatial navigation | | | Tarsitano & Jackson 1994 | |  |
| Dandy jumping spider (*Portia schultzi*) | detour-choice task | initial | | locomotion | | | void | | n/a | | spatial navigation, route planning | | | Cross & Jackson 2016 | |  |
| Dried banana leaf spider (*Neobrettus nangalisagus*) | detour-choice task | initial | | locomotion | | | void | | n/a | | spatial navigation, route planning | | | Cross & Jackson 2016 | |  |
| Fringed jumping spider (*Portia fimbriata)* | detour-choice task | continuous | | locomotion | | | void | | n/a | | spatial navigation | | | Tarsitano & Jackson 1994 | |  |
|  |  | initial | | locomotion | | | void | | n/a | | spatial navigation, route planning | | | Tarsitano & Jackson 1997,  Cross & Jackson 2016 | |  |
| Scorpion mimicking jumping spider (*Brettus albolimbatus*) | detour-choice task | initial | | locomotion | | | void | | n/a | | spatial navigation, route planning | | | Cross & Jackson 2016 | |  |
| White-moustached portia (*Portia labiata*) | detour-choice task | continuous | | locomotion | | | void | | n/a | | spatial navigation | | | Tarsitano & Andrew 1999, Tarsitano 2006 | |  |
|  | detour-choice task | initial | | locomotion | | | void | | n/a | | spatial navigation, route planning | | | Cross & Jackson 2016 | |  |
| *Brettus adonis* | detour-choice task | initial | | locomotion | | | void | | n/a | | spatial navigation, route planning | | | Cross & Jackson 2016 | |  |
| *Cocalus gibbosus* | detour-choice task | initial | | locomotion | | | void | | n/a | | spatial navigation, route planning | | | Cross & Jackson 2016 | |  |
| *Cyrba ocellata* | detour-choice task | initial | | locomotion | | | void | | n/a | | spatial navigation, route planning | | | Cross & Jackson 2016 | |  |
| *Cyrba simoni* | detour-choice task | initial | | locomotion | | | void | | n/a | | spatial navigation, route planning | | | Cross & Jackson 2016 | |  |
| *Gelotia lanka* | detour-choice task | initial | | locomotion | | | void | | n/a | | spatial navigation, route planning | | | Cross & Jackson 2016 | |  |
| *Meleon solitaria* | detour-choice task | initial | | locomotion | | | void | | n/a | | spatial navigation, route planning | | | Cross & Jackson 2016 | |  |
| *Portia africana* | detour-choice task | initial | | locomotion | | | void | | n/a | | spatial navigation, route planning | | | Cross & Jackson 2016 | |  |
| *Portia albimana* | detour-choice task | initial | | locomotion | | | void | | n/a | | spatial navigation, route planning | | | Cross & Jackson 2016 | |  |
| *Portia cf occidentalis* | detour-choice task | initial | | locomotion | | | void | | n/a | | spatial navigation, route planning | | | Cross & Jackson 2016 | |  |
| Other | | | | | | | | | | |  |  |  |  |  |  |
| Robot | four-compartment box | initial | | locomotion | | | semitransparent | | I | | problem solving in artificial systems | | | Walker & Miglino 1999 | |  |
